# The Strategic Council for Research Excellence, Integrity, and Trust

**DOI:** 10.1073/pnas.2116647118

**Published:** 2021-10-05

**Authors:** Marcia McNutt, France A. Córdova, David B. Allison

**Affiliations:** ^a^President, National Academy of Sciences;; ^b^President, Science Philanthropy Alliance;; ^c^Dean, Indiana University School of Public Health-Bloomington

## Abstract

We announce the creation of a new body within the National Academies of Sciences, Engineering, and Medicine called the Strategic Council for Research Excellence, Integrity, and Trust, charged with advancing the overall health, quality, and effectiveness of the research enterprise across all domains that fund, execute, disseminate, and apply scientific work in the public interest. By promoting the alignment of incentives and policies, adoption of standard tools, and implementation of proven methods, the Strategic Council seeks to optimize the excellence and trustworthiness of research for the benefit of society.

The research enterprise is vast and complex, and it has also become essential to health, prosperity, and national security in the 21st century. The current system grew up organically from modest beginnings in the 20th century, with the contributions of science to victory in the World Wars prompting a remarkable growth in peacetime to nearly half a trillion dollars of support annually from government, industry, and philanthropic sources in the United States alone. As with any complex human undertaking, the rapid growth has not necessarily resulted in maximum effectiveness and coordination among the various stakeholders, including funders, research institutions, researchers, publishers, and those who use research results for policy and other applications. Thus, researchers are confronted with a dizzying array of different requirements and policies, all roughly intended to ensure the excellence and integrity of research but time consuming, frustrating to satisfy, and not all obviously ensuring the excellence and integrity of research in actuality.

Several reports from the National Academies of Sciences, Engineering, and Medicine (NASEM) have noted the lack of any organization charged with strengthening capacity to support research excellence across all domains. Two reports, *Optimizing the Nation’s Investment in Academic Research* (2016) (1) and *Fostering Integrity in Research* (2017) (2), recommended the creation of a new entity focused on anticipating threats to research integrity and streamlining and improving accountability throughout the research enterprise. Yet, given the decentralized nature of research involving government, industry, and nonprofit organizations, it was not clear who could step up to this task. To be successful, any willing entity would need to have the respect of the scholarly community and public, understand the challenges encountered in all aspects of research, and be minimally conflicted to avoid having an agenda tuned to its own interests.

A group of stakeholders across all facets of the research enterprise convened for a retreat at the Annenberg Retreat at Sunnylands and recommended that the National Academy of Sciences (NAS) should step up to this mandate and engage the National Academy of Engineering (NAE) and National Academy of Medicine (NAM) in partnership. This recommendation furthermore aligns with two of the objectives in the Strategic Plan of the NAS: “Promote excellence and diversity in the scientific workforce” and “Support the basic research enterprise across all disciplines.” Further conversations within the Academies resulted in the Committee on Science, Engineering, and Medicine for Public Policy (COSEMPUP) agreeing to be the parent body for a “Strategic Council for Research Excellence, Integrity, and Trust.” The concept of a Strategic Council (SC) is new for the Academies. This body will function in a mode with similarities to both a committee and a roundtable. Like a roundtable, the SC will include stakeholders across the research enterprise, although none are appointed by any sponsors of the SC. Like a committee of the Academies, the SC can discuss, originate, and disseminate best practices, request creation of study committees to issue consensus reports on key issues, and form action collaboratives to implement recommendations from such reports.

The charge to the SC is 1) identifying, anticipating and prioritizing key challenges to research ethics, integrity, and trustworthiness; 2) articulating principles, policies, and best practices to address them; 3) catalyzing progress by coordinating collaborative action; and 4) breaking barriers where needed to accelerate solutions.

The inaugural operations of the SC are being supported by the NAS and a grant from the Gordon and Betty Moore Foundation.

## Potential Topics to Be Addressed

The SC has already assembled an expansive list of potential topics to address such as addressing inefficiencies, aligning incentives, and promoting excellence, transparency, and trust. We describe just a few examples of how the SC might help.

### Conflict of Interest.

Current processes for declaring an author’s relationships that might be viewed as potentially impacting his or her ability to address an issue without bias* are cumbersome, uneven, and inefficient. Researchers are asked to make multiple independent declarations of outside interests and commitments to their institutions, funders, and journals each year. Commonly such declarations are left to the judgment of the individual as to which items are relevant, and there is no consensus as to what needs to be declared. Current relationships only? Past and future commitments? Holdings and activities of family members or mentors or close collaborators? International awards or recognitions? It is no wonder that when researchers are occasionally tripped up for not having declared a certain relationship the most common response is “I didn’t think I needed to declare that.” Of the many issues that the SC could take on, this one might be one of the easier ones to address. Already researchers have up-to-date publication lists linked to their ORCID. A possible extension of this practice would be for researchers to continually update their lists of current and prior employment, activities, commitments, and holdings, linked again to an ORCID. These files would be viewable by any user of that researcher’s work, and the user could decide which relationships might have influenced the work at the time it was completed. To reduce burden and complexity in applying for federal grants all science agencies would need to agree on a universal format and content.

### Assessment of Researchers.

Here we discuss rewards for scientists in academia, but we note that academia now employs fewer than half the scientists in the United States (3). The academic reward system requires assessment of researchers. It is exceptionally difficult to devise approaches that catalyze exiting, bold research efforts and lead, in the long run, to exciting, novel, and importantly useful findings, are simultaneously easy to implement, equitable, and useful measures of excellence, and have only negligible deleterious side effects in addition to their positive intended effects. Current assessment methods and rewards systems are widely criticized for their unintended consequences and frequently labeled “perverse incentives.” For example, the practice of counting numbers of publications has led to such undesirable outcomes as the proliferation of incremental research publications (the “least publishable unit”) and the uptake of predatory journals that provide a publication outlet without the need to pass muster from peer review. On the other hand, the practice of putting greater weight on publications in high-impact journals encourages authors to selectively put effort into sensational findings, while overlooking the importance of publishing reproducibility of the findings. Even counting citations as a measure of research impact has numerous drawbacks: Citation rates are field and subfield dependent (but also notable strengths). A researcher truly ahead of his or her time might not see papers cited until a decade or more after publication. Some creative solutions are being implemented to address the unintended consequences, yet citation counts and h-indices have their strengths (4) and advocates (5), and we should not throw the baby out with the bathwater. A few funding agencies are asking principal proposers to select a few of their contributions and document their impact. Some research institutions are similarly putting more emphasis on the impact than on numbers of publications or venues. Assessing the success of these approaches (and any unintended consequences) would be a valuable undertaking for the SC, with the goal of more broadly disseminating useful solutions to assessment.

### Retractions.

Correcting the literature is an important part of the self-correcting nature of science. Unfortunately, there is little agreement on how and when to retract a study, as well as unwanted stigma attached to retractions even when the error was an honest mistake. Furthermore, the act of retraction generally involves cooperation between research institutions (most able to investigate errors or misconduct) and journals which need to correct the record; one or both of these parties may not see it in their own best interests reputationally to cooperate. Most agree that retracting very old papers that might simply be outdated is a waste of resources, when an entire field has moved on to more modern methods and interpretations. However, there is a fuzzy line in deciding which older papers should still be retracted because they are having undue influence on science policy, even if the scientific community has rejected the older data or interpretation. Finally, there are conflicting goals in retraction. Is the goal to prevent the flawed work from being disseminated? Or is it to discourage careless or fraudulent research? An approach that maximizes the speed for journals to correct the record is more likely to be viewed as being nontransparent on what actually went wrong (e.g., misconduct because data were fabricated versus simple error in using incorrect instrument calibration) and likely punitive to some coauthors who might not have played any role in the errors. A number of authors have suggested alternative approaches to retractions (6–10), but as of yet there has been insufficient momentum, leadership, and coordination to improve the practice (11). Better approaches to retractions could be a promising opportunity for the SC to contribute to research excellence.

There are many additional important issues the SC will want to address. Public trust in science is high on this list. The formation of the SC is based on the premise that science would be better served with an increased, collaborative emphasis on integrity of the research process and research culture and accountability of researchers. The SC will work toward these goals.

## Membership

The three authors of this contribution (M.M., F.A.C., and D.B.A.) have agreed to cochair this activity. A distinguished group of additional leaders across the research sectors have volunteered their valuable time, experience, wisdom, and expertise to launch this undertaking (Table 1). The SC represents funders of science, leaders from research institutions, publishers who disseminate research findings, and users of sound scientific information, as well as some leaders from the international research community to provide perspective.

**Table 1.** Members of the SC for Research Excellence, Integrity, and Trust (cochairs shaded in gray)

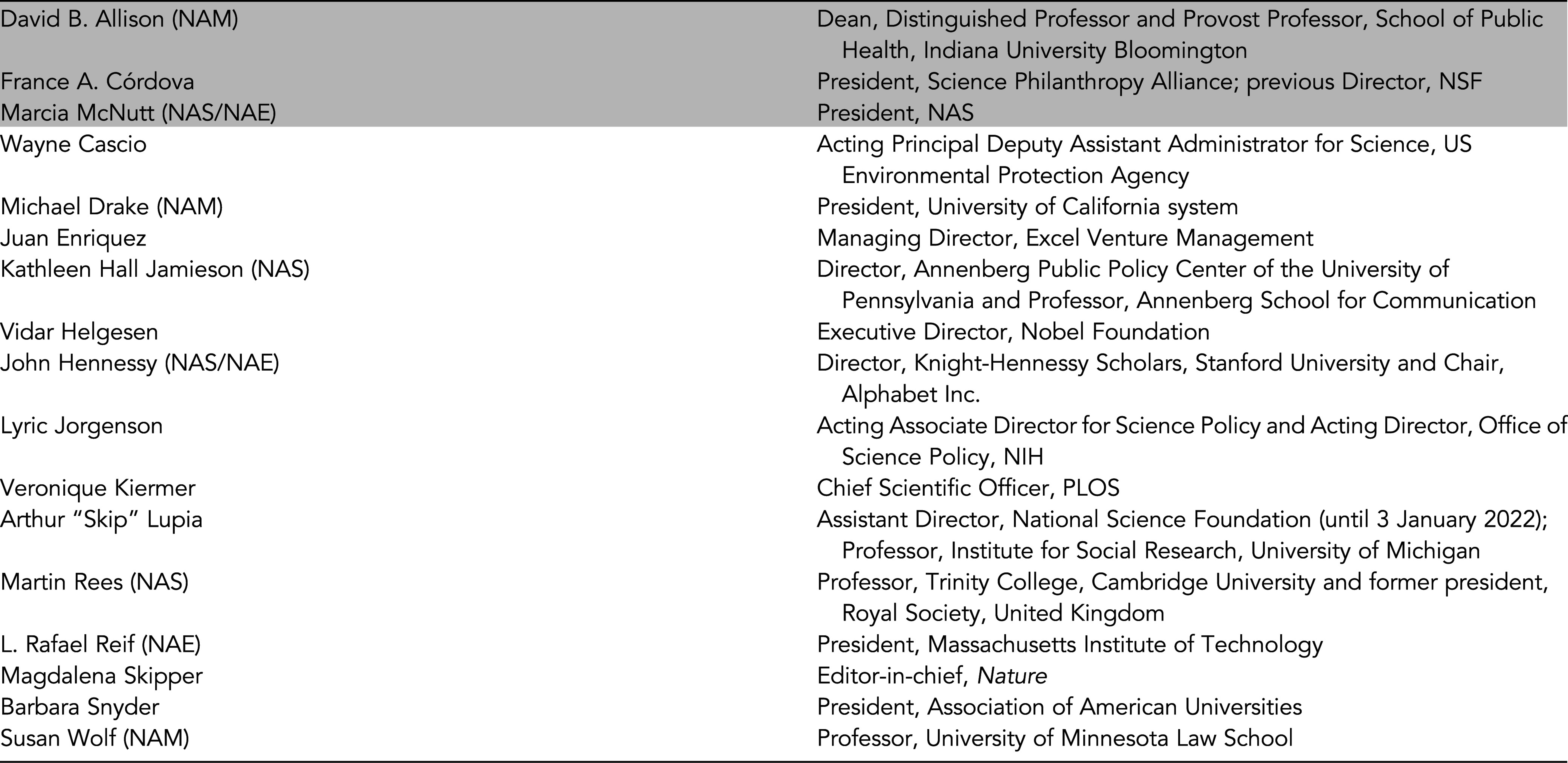

At the SC’s first meeting in October, the inaugural members will discuss potential gaps in expertise and representation needed for the SC to execute its task.

## Conclusion

By anticipating, analyzing, and addressing challenges to the excellence of the research enterprise the SC can become a catalyst for greater trust, integrity, and transparency in research. The SC will look for opportunities to gain agreement across the many stakeholders in the research enterprise as to what changes should be broadly supported to achieve alignment of incentives, quality, and efficiency. By encouraging the creation of new tools, the SC can make it easy for researchers to know what is expected of them and make it easier to meet those expectations. We look forward to hearing from members of the research community as to important topics affecting the trust and integrity of research that are candidates for the SC to address.

## Footnotes

*The very subjectivity in what even constitutes bias is sobering in this context. In some domains, e.g., inferential statistics, we have precise objective mathematical definitions of bias [see C. Rao, Sufficient statistics and minimum variance estimates. *Math. Proc. Cambridge Philos. Soc*., **45**, 213–218 (1949); doi:10.1017/S0305004100024737]. But, in this more subjective way, “bias” requires subjective judgments about fairness or reasonableness and inferences about persons’ states of mind as typified by this definition of bias from the *Cambridge Dictionary*: “the action of supporting or opposing a particular person or thing in an unfair way, because of allowing personal opinions to influence your judgment.” Thus, honest, intelligent people can disagree about what relationships are potentially biasing, relevant, or disclosure-worthy [see J. Ioannidis, Conflict of Interest in Nutrition Research—Reply. *JAMA*, **320** 94–95 (2018). doi:10.1001/jama.2018.5678].
